# Contribution of Host Defence Proteins and Peptides to Host-Microbiota Interactions in Chronic Inflammatory Lung Diseases

**DOI:** 10.3390/vaccines6030049

**Published:** 2018-07-28

**Authors:** Anne M. van der Does, Gimano D. Amatngalim, Bart Keijser, Pieter S. Hiemstra, Remi Villenave

**Affiliations:** 1 Department of Pulmonology, Leiden University Medical Center, Leiden 2300 RC, The Netherlands; p.s.hiemstra@lumc.nl; 2 Department of Pediatric Pulmonology, Wilhelmina Children’s Hospital, University Medical Center Utrecht, Utrecht 3508 AB, The Netherlands; g.d.amatngalim@umcutrecht.nl; 3 Regenerative Medicine Center, University Medical Center Utrecht, Utrecht 3508 AB, The Netherlands; 4 Research Group Microbiology and Systems Biology, TNO (The Netherlands Organization for Applied Scientific Research), Zeist 3704 HE, The Netherlands; bart.keijser@tno.nl; 5 Department of Preventive Dentistry, Academic Center for Dentistry Amsterdam (ACTA), University of Amsterdam, Amsterdam 1008 AA, The Netherlands; 6 Emulate Inc., Boston, MA 02210, USA; remivillenave@gmail.com

**Keywords:** chronic inflammatory lung diseases, COPD, Asthma, cystic fibrosis, microbiota, host defence peptides, host-microbiota interactions

## Abstract

The respiratory tract harbours a variety of microorganisms, collectively called the respiratory microbiota. Over the past few years, alterations in respiratory and gut microbiota composition have been associated with chronic inflammatory diseases of the lungs. How these changes influence disease development and progression is an active field of investigation. Identifying and understanding host-microbiota interactions and factors contributing to these interactions could promote the development of novel therapeutic strategies aimed at restoring host-microbiota homeostasis. In this review, we discuss recent literature on host-microbiota interactions in the respiratory tract, with a specific focus on the influence of endogenous host defence peptides and proteins (HDPs) on the composition of microbiota populations in vivo and explore possible HDPs-related therapeutic approaches targeting microbiota dysbiosis in chronic inflammatory lung diseases.

## 1. Introduction

Despite our knowledge of the bacterial abundance in the gut, it was only during the last decade that scientists fully recognised the crucial role played by the microbiota in human health and disease [1]. This development was spurred mainly by the availability of new techniques that allowed highly sensitive and culture independent detection of both aerobic and anaerobic microbial species [2]. Not only the gut, but also the skin, lungs and many other sites of the human body turned out to be a natural habitat for a variety of microorganisms [2]. Besides the by now well-described bacterial population of these sites, evidence for viral and fungal presence and interactions were also established, and are referred to as the virome [3,4,5] and mycobiome [6], respectively. Collectively these organisms compose the microbiota, although this specific term is generally used to describe solely the bacterial component, since this has so far received most attention. 

Studies have revealed a spatial heterogeneity of the respiratory microbiota, with the composition of bacterial populations depending on the anatomical location from which samples were derived, e.g., nasal, tracheal, bronchial or alveolar [7,8,9,10]. Furthermore, the respiratory microbiota varies in composition between healthy individuals but also between healthy individuals and patients suffering from chronic inflammatory lung diseases such as asthma [11], chronic obstructive pulmonary disease (COPD) [9] and cystic fibrosis (CF) [12]. Our current knowledge of host-microbiota functional interactions and dynamics in the lungs is very limited. For instance, it is still unclear whether changes in microbiota composition contribute to disease pathology or if chronic diseases of the lungs drive microbiota disbalance. Discovery and characterisation of microbiota variability in health and disease, combined with studies on how the reported changes influence disease pathology provided the rationale for a new field exploring novel therapeutic strategies aiming at restoring balance in microbiota composition. However, to successfully modulate the respiratory microbiota in chronic respiratory lung diseases, it is essential to understand local host-microbiota interactions and identify host factors that contribute to changes in microbiota composition.

This review will discuss the latest advances in respiratory host-microbiota interactions with specific focus on host defence proteins and peptides (HDPs). These conserved antimicrobial molecules, which include but are not limited to the family of cationic antimicrobial peptides, are expressed by all human body sites that interact with microbiota and display broad-range direct and indirect activities against bacteria, viruses, and fungi, and may therefore provide interesting therapeutic possibilities [13].

### Chronic Inflammatory Lung Diseases

During the past few years, studies revealed alterations of microbiota composition in patients with chronic inflammatory lung diseases. Here we will specifically discuss these differences in the context of three chronic pulmonary inflammatory diseases, namely chronic obstructive pulmonary disease (COPD), asthma and cystic fibrosis (CF).

Patients with COPD generally suffer from an individual-specific combination of two diseases: obstructive airway disease (chronic bronchitis) and emphysema [14]. COPD patients with chronic bronchitis suffer from chronic inflammation of the larger airways resulting in airway wall remodelling and sustained over-production of mucus (related to the typical smokers’ cough), while individuals with emphysema experience a progressive decline of lung function due to the destruction of the specific 3-dimensional architecture of the alveolar tissue. In addition, COPD patients suffer from small airway disease (SAD), suggested to be the site of initiation of COPD [15], leading to increase in small airway resistance [16]. In conjunction with tissue damage and remodelling, respiratory tract infections can trigger disease exacerbations and aggravate disease progression in these patients.

The main risk factor for COPD in industrialised countries is cigarette smoke, nevertheless air pollution caused by indoor cooking and heating for instance in poorly ventilated homes in less affluent countries also contributes significantly to disease development. COPD severely reduces the quality of life of patients, affecting millions of people and is currently the third leading cause of mortality worldwide [17]. To this day, no therapies are available to cure or reverse pathology, and current treatments are limited to reducing symptoms mainly by promoting smoking cessation and by use of glucocorticoids and bronchodilating β2-agonists and muscarinic receptor antagonists [14]. 

With approximately 300 million people affected worldwide, asthma has a significant contribution to chronic obstructive pulmonary diseases. Asthma has a highly heterogeneous pathology with the largest portion of patients displaying chronic type-2 inflammation in their airways characterised by influx of eosinophils, smooth muscle hypertrophy and goblet cell hyperplasia [18]. Airway hyperreactivity, contraction of airway smooth muscles and increased mucus production results in narrowing and obstruction of the airways leading to severe shortness of breath. In addition to the Th2-high asthma in which allergen exposure may be an important trigger, patients can also present with non-allergic/non-atopic asthma. Other asthma endotypes include those triggered for example by exercise [19] or aspirin [20], while asthmatics may also present with mixed features of both asthma and COPD (asthma COPD overlap syndrome, or ACOS) [21]. Although they have many similarities, these manifestations of asthma are increasingly regarded as separate phenotypes [22]. Like COPD, respiratory infections can also trigger disease exacerbation that contribute to worsening of asthma symptoms. Furthermore, there is no curative treatment for asthma with only symptom-alleviating medications available for patients. Several new drugs have recently entered the market, mainly for severe asthma patients in which regular treatment with anti-inflammatory glucocorticoids or bronchodilating β2 agonists offers insufficient disease control [23].

CF is an autosomal recessive inherited disease affecting several organs but particularly devastating to the lungs, caused by mutations in the cystic fibrosis transmembrane conductance regulator (CFTR) gene [24]. The CFTR protein mediates anion secretion at the surface of airway epithelial cells and thus regulates the volume of the periciliary layer [25]. As a consequence of attenuated expression, folding or function of the CFTR protein, patients with CF suffer from microbial colonisation and infections that may result from impaired host defence resulting from altered composition of these airway secretions [26]. This is associated with neutrophilic airway inflammation and progressive lung tissue remodelling, which are observed from a young age [27]. Treatment of CF lung disease is largely focused on promoting airway clearance and reducing bacterial outgrowth with antibiotics [28]. However, recently developed small molecules improving folding or gating of the defective CFTR protein, referred to as correctors and potentiators respectively, allow target-specific treatment of certain patients [29].

Taken together, these chronic lung diseases contribute substantially to worldwide disease burden and mortality with little to no curative treatments available. Furthermore, all three diseases are characterised by increased frequency of respiratory infections, contributing to a worsening of symptoms, referred to as exacerbation or lung attack. These exacerbations frequently result in hospitalisation and may in some patients be lethal. Additional therapeutic approaches are therefore needed to reduce the risk of exacerbations in these patients, prevent disease progression and ultimately improve quality of life.

**Figure 1:**
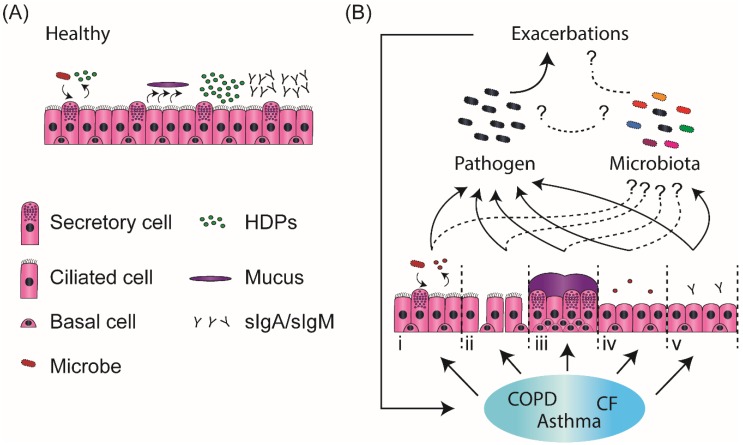
Summary of processes that contribute to the increased frequency of respiratory infections in patients with chronic inflammatory lung disease. The healthy epithelium exerts an array of host defence activities that contribute to the prevention of pathogen invasion: it forms a tight and physical barrier, secretory cells that include club cells and goblet cells contribute to the production of host defence molecules such as host defence peptides and proteins (HDPs), ciliated cells in combination with the mucus produced by the goblet cells promote mucociliairy clearance, and transport of IgA and IgM across the epithelium allows sufficient secretory IgA/IgM (sIgA/sIgM) to be present in the lumen to contribute to immune exclusion (**A**). In patients with chronic obstructive pulmonary disease (COPD), asthma, or cystic fibrosis (CF) several of these processes are disturbed and contribute to an enhanced frequency of respiratory infections: (i) Chronic inflammation alters epithelial responses to pathogens through impaired cytokines/chemokines secretion and decreased HDPs secretion and/or activity. (ii) Chronic inflammation leads to impaired barrier function of the airway epithelium allowing bacterial invasion of the airways. (iii) Changes in mucus secretion, composition and rheology as well as goblet cells metaplasia in a chronically inflammed epithelium leads to impaired mucociliary clearance, resulting in outgrowth of potenial pathogenic bacteria. (iv) Airway epithelial remodelling can lead to the reduction of HDP-producing cells, facilitating bacterial colonisation. (v) IgA/IgM transport across the airway epithelium to the mucosal secretions may be impaired as a result of changes in pIgR expression during chronic inflammation (**B**). While these processes have been shown to affect pathogen invasion and growth, it is still unclear how they impact microbiota composition and further increase respiratory infection risks.

Several factors are suspected to contribute to the increased frequency of respiratory infections/exacerbation in these patients: (i) alteration of the host immune response to respiratory pathogens through modulation of cytokines/chemokine secretion [30] and decreased production and/or activity of epithelial HDPs [31]; (ii) epithelial injury induced by chronic inflammation or direct exposure to cigarette smoke exposure, resulting in, e.g., decreased mucosal integrity and barrier activity [32]; (iii) airway epithelium remodelling resulting in impaired ciliary activity and modification of mucus secretion, composition and physical properties leading to altered mucociliary transport and deficient pathogen clearance [33,34,35,36,37,38]; (iv) changes in airway epithelial cell composition negatively affecting the presence of host defence proteins and peptides (HDPs) and leading to locally impaired antimicrobial defences [39]; (v) IgA/IgM transport across the airway epithelium into mucosal secretions may be impaired as a result of changes in pIgR expression in these diseases [40,41]. These processes and how they are affected in chronic inflammatory lung diseases are summarised in Figure 1. While the link between these processes and airway infections has clearly been established, their contribution to the microbiota at this point still remains understudied.

## 2. Respiratory Microbiota Composition in Health and Disease

### 2.1. The ‘Healthy’ Respiratory Microbiota

The existence of a bacterial population stably colonising the lungs has been a matter of (intense) debate. However, it is now clearly established that each individual possesses a dynamic population of aerobic and anaerobic bacteria [42]. The composition and the abundance of the respiratory microbiota composition differs between the different anatomical sections of the respiratory tract [8,43]. For instance, the bacterial population present in the nasal cavity differs in composition from the larger and smaller airways. Similarly, the oral microbiota is also distinct from the nasal cavity [10]. Several studies have furthermore demonstrated that the airway microbiota shows more resemblance with the oral than the nasal microbiota, suggesting that microaspiration of saliva contributes to microbiota composition in the lungs [8,44]. However, it was also hypothesised that contamination during the sampling process might explain these findings. To elucidate the origin of the lung microbiota, Pragman and co-workers [45] assessed microbiota composition in lung tissue obtained by lobectomy, excluding the risk of carry-over from the oral or nasal cavity and showed that the lung microbiota of COPD patients contained both typical nasal and oral microbiota bacteria, thus concluding that aspiration indeed was a source for microbiota in the lower respiratory tract [45]. 

The bacterial component of the microbiota is a combination of non-pathogenic, commensal bacteria strains and potentially pathogenic bacteria strains, also called pathobionts [43]. The major bacterial phyla detected in the oral cavity are: Bacteroidetes, Firmicutes, Proteobacteria, and Fusobacteria. At genus level, great variation is observed depending on the type of surface colonised, such as teeth or soft mucosal tissues. Typical bacterial genera identified include *Streptococcus*, *Veilonella*, and *Prevotella* spp. The nasal cavity harbours bacteria from the phyla *Firmicutes*, *Proteobacteria*, *Bacteroidetes*, and *Actinobacteria* with typical bacterial genera being *Corynebacterium*, *Moraxella*, *Dolosigranulum*, *Streptococcus* and *Staphylococcus* spp. [43,46,47]. The major phyla of the respiratory tract include Firmicutes, Bacteroidetes and Proteobacteria [9,48], with the most prevalent genera those of *Pseudomonas*, *Streptococcus*, *Prevotella*, *Fusobacterium* and *Veillonella* [9,49].

To date, little is known about the composition of the virome in healthy adults. The limited studies performed show that both the oral cavity and the respiratory tract display high levels of bacteriophages (mainly Siphoviridae, Myoviridae and Podoviridae) [50]. It was also observed that the virome composition is more complex in children with severe acute respiratory infections when compared with children without these infections [51]. The mycobiome in the respiratory tract is comprised of both filamentous and spore forming fungi. Typical phyla described in studies are Ascomycota and Basidiomycota [52]. In healthy subjects the fungal contribution to the microbiota is characterised mainly by environmental agents such as *Davidiellaceae* and *Cladosporium* and low levels of *Aspergillus* [53], but its composition is markedly affected by disease [54].

### 2.2. The ‘Diseased’ Respiratory Microbiota

Differences in microbiota composition in chronic inflammatory lung disease patients have been reported by multiple research groups. While smokers display significant changes compared to healthy individuals with regard to their oral and nasal microbiota, their respiratory microbiota is relatively unchanged by smoking per se, but rather changes in relation to lung function decline [9,49]. The composition of the lung microbiota in COPD patients is however strongly altered, with larger proportion of Firmicutes [55] or Proteobacteria (usually associated with bacterial exacerbations) and less Bacteroidetes than in healthy individuals [55]. These observed differences seem to correlate with disease progression [10,56]. The main genera found in the lungs of COPD patients are: *Streptococcus*, *Corynebacterium*, *Alloiococcus*, *Prevotella*, *Veillonella*, *Rothia*, *Neisseria*, and *Staphylococcus*. By comparing 16S rRNA gene copies, Pragman et al. furthermore showed that bacteria were less abundant in the lower than in the upper respiratory tract. The microbial population in the lower respiratory tract was more diverse than in the oral or nasal cavity of individuals with COPD [45]. The bacterial composition of bronchial tissue and peripheral tissue however, was highly similar [45]. Mayhem and co-workers furthermore showed that during exacerbations the microbiota is severely impacted and that the type of exacerbations these patients suffered from (mainly bacteria-driven exacerbations or mainly eosinophilic exacerbations) influenced the composition of the microbiota [56]. Interestingly, recently it was shown that lung extracellular vesicles also contained bacterial communities, distinct from the epithelium-associated microbiota [57]. Despite identification of changes in the lung microbiota composition of COPD patients, little is known about the variation in mycobiome and virome composition.

Alteration of microbiota composition in asthma was also suggested to contribute to the disease development. Already during childhood, changes in microbiota composition are associated with asthma development [58,59]: for instance, gut microbiota diversity has been related to asthma development. A study demonstrated that in one-year-old children from asthmatic mothers the immaturity of the microbiota was associated with asthma development at later age [59]. This was further supported by studies showing that development of asthma was affected by the diversity of the microbial communities children were exposed to [60] and respiratory colonisation with typical opportunistic pathogenic genera such as *Moraxella* or *Haemophilus* early in life were associated with a higher risk to develop asthma [61].

Overall, patients with asthma have a distinct respiratory microbiota from healthy individuals with increased Proteobacteria and reduced Bacteroidetes levels [11,62,63]. Indeed, Taylor et al. showed that neutrophilic asthma was associated with high abundance of *Haemophilus* and *Moraxella*, whereas abundance of *Gemella*, *Porphyromonas* and *Streptococcus* taxa correlated negatively with this phenotype [64], suggesting that specific bacteria in the microbiota could be associated with specific asthma phenotypes such as neutrophilic asthma versus eosinophilic asthma. Furthermore, Huang et al. showed that specific genera were associated with clinical features of severe asthma [62]. A negative correlation was found between eosinophil levels and presence of Proteobacteria and specific members of the Firmicutes genera, while a higher bacterial burden was associated with less eosinophils. Interestingly, results showed that in severe asthma abundance of *Klebsiella* was highly increased.

Alterations in the respiratory microbiota in individuals with CF have already been observed during the first months after birth. This was shown in a study comparing the nasopharyngeal microbiota from CF infants with non-CF control subjects, which demonstrated early colonisation of *Staphylococcus aureus* and a lack of typical commensal microbes, such as *Corynebacterium pseudodiphtericum* [65]. The lower respiratory microbiota of CF infants displays similarities with the oral- and nasopharynx, which might be due to microaspiration [66]. Furthermore, CF infants are characterised by having a diverse and dynamic respiratory microbiota with large inter-patient variation. This is in contrast to adult CF patients, which display a less diverse respiratory microbiota, predominately characterised by colonisation with *Pseudomonas* or *Burkholderia* [67,68]. In addition, significant differences in mycobiome composition have been recorded for CF patients. A study involving 89 CF patients showed that almost half of them were colonised with *C. albicans* in their lungs. Often this was accompanied by co-localisation with *Pseudomonas* and was related to exacerbation frequency and FEV_1_ decline [69]. An additional study showed that CF patients colonised with *A. fumigatus* displayed significantly lower FEV_1_ values [70].

## 3. HDPs Contributing to Microbiota Composition

According to Dickson et al., the composition of the respiratory microbiota is maintained through immigration, regional growth conditions and elimination [71]. As discussed above, immigration is partly dependent on microaspiration and on inhalation. Elimination is mediated by a combination of processes including the previously mentioned mucociliary clearance/cough mechanism. Regional growth conditions include factors such as local pH, nutrients, oxygen and salt levels and locally produced mediators including HDPs. In lung diseases, the contribution of these three processes shift from a large influence of immigration and elimination in healthy individuals to a larger contribution of regional growth conditions in patients with chronic inflammatory lung diseases, especially during exacerbations [72]. In this review, we particularly discuss the possible role of HDPs in these growth conditions.

### 3.1. Host Defence Peptides/Proteins in the Airways

The airway epithelium has a vast array of defence mechanisms at its disposal to prevent colonisation or infection by pathogenic microorganisms [73]. HDPs are natural antimicrobials synthesised by the body, highly conserved among species and important for a variety of host defence functions [74]. Here, we use the abbreviation HDP as a collective name for peptides and proteins with direct antimicrobial activity (antimicrobial peptides or AMPs) and with immunomodulatory functions (generally called HDPs). HDPs are often small, cationic molecules the expression of which can be induced by a variety of triggers, including bacterial, viral or fungal-derived components [75] and regulated by processes such as inflammation, tissue repair, ER stress [76] or vitamin D [77,78]. Other HDPs are constitutively expressed and therefore part of the airway epithelial barrier during homeostasis [79,80,81]. 

HDPs have a variety of direct and indirect antimicrobial functions that include prevention of growth or killing of bacteria, viruses and fungi (reviewed in [82,83]) or anti-biofilm activity [84]. HDPs can also regulate immune responses through influencing immune cell recruitment [85] or promoting phagocytic capabilities of immune cells [86] leading to microbial clearance. Depending on their mechanism of action their activity can be broad-spectrum or pathogen specific. Their function can also extend beyond host defence activity as some HDPs also for instance influence angiogenesis [87] or display antitumor activity [88]. It was furthermore shown that HDPs are not only essential for activities against pathogenic microorganisms but can also contribute to microbiota composition in the gut [89].

Both airway epithelial and immune cells produce a large array of HDPs. Typical HDPs such as defensins and cathelicidins (humans express only one cathelicidin named hCAP18/LL-37) have been extensively studied and are well characterised. Expression of HDPs can be cell-type specific; for example, human alpha defensins are expressed by neutrophils (HNP1-4) or Paneth cells (HD5 and HD6), whereas LL-37 is much more widely expressed by both epithelial cells [75] as well as various types of immune cells [85]. However, the respiratory epithelium produces an additional array of peptides and proteins that are important for host defence, including (but not limited to) lactoferrin, secretory leukocyte proteinase inhibitor (SLPI), short and long palate, lung and nasal epithelium clone protein (PLUNC), lipocalin 2, S100A7 and RNase7 (reviewed in [73]). Most of these latter mentioned HDPs are highly expressed during homeostasis and may therefore actively participate to barrier function of the airway epithelium. Their high expression at the airway epithelial site during homeostasis suggests that they could furthermore contribute to microbiota interactions. In the gut and on the skin, it has already been shown that HDPs indeed contribute to microbiota composition. For instance, mice engineered to express human alpha defensin 5 (HD5) [90] or mice that were deficient for matrilysin (the processing enzyme for murine defensin) displayed a changed composition of the gut microbiota when compared to wild-type mice [89]. In vitro hBD-2 and -3 showed selectivity in antimicrobial activity against pathogenic and commensal strains when tested with a gut epithelial cell line transfected to express these hBDs [91]; this selectivity was also demonstrated for the murine α-defensin cryptdin-4 [92]. This observation is in line with another study demonstrating that in the gut, commensal bacterial strains showed a degree of resistance to inflammation-related HDPs whereas pathogenic strains did not [93].

A further role in the contribution of defensins to microbiota composition was suggested following the observation of an association between reduced defensin copy number and differences in nasopharyngeal bacterial colonisation patterns [94]. Besides defensins, RegIIIɣ—an HDP that is highly produced in the gut—was demonstrated to be essential for microbiota control. The presence of this HDP in the gut of mice created a microorganism-free zone between the microbiota and the gut epithelial cells [95]. In addition to providing control of the microbiota by direct interaction with human-derived HDPs, recently it was shown that human HDPs can also affect the microbiota by synergising with bacteria-derived antimicrobials to selectively kill strains that are part of the (skin) microbiota [96].

Finally, HDPs may modulate inflammation induced by microbial exposure as HDPs affect several TLR-mediated immune responses to bacteria, viruses and fungi [97] suggesting another mechanism by which HDPs may contribute to the regulation of the composition of microbiota.

### 3.2. Changed HDP Activity and Expression in Chronic Inflammatory Lung Diseases

Studies have so far shown a role for HDPs in microbiota composition, while other studies demonstrated how their expression and/or activity can be impaired in chronic inflammatory lung diseases. Consequently, changes in microbiota composition found in chronic lung diseases could (partly) be the consequence of changed HDP expression or activity in these diseases. For instance, local vitamin D metabolism was demonstrated to be disturbed during chronic inflammation of the airways, with reduced host defence as a consequence [98]. In addition, cigarette smoke exposure has a marked effect on expression of HDP expression by airway epithelial cells [31,99]. Disease-specific features have been reported, as illustrated by the decreased expression of selected HDPs by cultured airway epithelial cells from COPD patients [31]. Recently we showed that several HDPs that are highly expressed during homeostasis, are expressed in a cell-specific manner by the epithelium and therefore airway remodelling may negatively impact their expression, leading to selective impairment of airway epithelium antimicrobial activity [39]. These data also suggest that altered HDP expression by remodelled epithelium could potentially affect microbiota composition in COPD, asthma and CF. Furthermore, changes in mucus composition that occur in these diseases can impair functionality of HDPs as their activity is dependent on local salt concentration [100] and can be inhibited by specific (serum) proteins [101].

In CF, alterations of the physiological conditions of the airway surface liquid may impair the function of HDPs. A lowered airway surface liquid pH, due to impaired bicarbonate secretion by CFTR, may reduce the antibacterial activity of HDPs [102,103,104]. Moreover, acidic pH may abrogate the ENaC inhibitory function of SPLUNC1, contributing to mucus dehydration [105]. The antibacterial activity of HDPs can furthermore be blocked by accumulated mucus [106] or attenuated upon binding to DNA, F-actin and glycosaminoglycans [107,108], which may be relevant for not only CF, but also for HDP activity in COPD and asthma.

### 3.3. Induction of HDPs by the Microbiota

Consistent with the ability of several bacteria from the respiratory microbiota to alter HDP expression, changes in microbiota composition can also affect HDP expression, either directly or through indirect secretion of microbial products. In this regard, short chain fatty acids (SCFAs) have received a lot of attention as a mechanism for indirect control of HDP expression by the microbiota. SCFAs are produced by the gut microbiota upon fermentation of dietary fibers and were shown to have a significant impact on immune responses, including those in the lung, i.e., the gut-lung axis [109]. For example, Trompette et al. demonstrated that SCFAs from the gut affected local immune responses in the lung by reducing the capacity of DCs to promote Th2 proliferation by T cells [109]. SCFAs also promoted cathelicidin expression in colon epithelial cells [110], while it was demonstrating that their promoting effects on defensin and regIIIɣ expression was mediated by the SCFA receptor GPR43 [111]. While this has yet to be validated in vivo, these activities may possibly be further contributing to host-microbiota interactions. Additional effects by several SCFAs on cathelicidin expression in vitro in gut and lung epithelial cells have been demonstrated [112,113]. Direct effects of bacterial strains on HDP expression were also demonstrated with probiotic *lactobacilli* and *E. coli* Nissle strains that induced hBD-2 expression by gut epithelial cells [114,115]. On (murine) skin the presence of microbiota was also associated with increased expression of HDPs like SLPI [116,117]. The involvement of microbiota in regulating HDPs expression has been further suggested by a study showing a time dependent increase of HDPs in the guts of infants during gut colonisation [118]. An altered microbiota composition can therefore lead to dysregulated HDP expression, which possibly lead to a further change in microbiota composition via a positive feedback loop.

## 4. Possible Therapeutic Interventions

The role of HDPs in the control of host-microbiota homeostasis is becoming increasingly clear and simultaneously creates possible new therapeutic approaches for resolving microbiota disbalance. Modulation of airway epithelial expression of HDPs could be a valid approach. This is supported by observations in the rabbit and human gut showing that SCFAs are able to promote HDP expression [119,120]. While this mechanism has not yet been translated to the airways, it was confirmed in vitro for butyrate using an airway epithelial cell line [121]. Despite these potential positive effects of SCFAs, it was demonstrated that SCFA levels that were detected in the sputum of CF patients displayed in vitro detrimental effects with regard to inflammatory responses, bacterial growth [122] and phagocytic capacity of immune cells [123]. SCFAs might therefore affect airway biology differently from gut biology and its use for therapeutic effects on the lungs may preferentially lie in the gut by targeting the gut-lung axis.

Another strategy to alter local levels of HDPs could be the administration of vitamin D. In the respiratory epithelium vitamin D was shown to promote cathelicidin antimicrobial peptides (*CAMP*) expression, the gene encoding LL-37 [124]. Furthermore, in chronic inflammatory lung disease, low levels of vitamin D rendered the patient more susceptible for infection leading to exacerbation [125,126,127,128], possibly through inflammatory cytokines affecting local vitamin D metabolism resulting in reduced levels of local vitamin D [98]. To this point it has not been investigated if vitamin D administration could directly affect microbiota composition. However, preventing exacerbations through vitamin D administration could indirectly prevent changes in microbiota composition that could lead further inflammation and deterioration in the quality of life of the patient. It has yet to be established whether airway epithelial remodelling promotes microbiota differences, although studies—including our own [39]—highlight this possibility and therefore alternative strategies aimed at restoring airway epithelial composition in the airways of these patients may further restore host-microbiota homeostasis. 

Alternative therapeutic strategies include targeting the respiratory microbiota directly. For example, in mice [129,130] and in rats (CF model) [131] it was shown that *Pseudomonas aeruginosa* alone could be eliminated by endogenous overexpression of *CAMP* and by delivery of LL-37 to the lungs. As specific bacterial products have shown to affect HDP expression in the lungs, administration of probiotic or commensal strains could change the dynamics of the respiratory microbiota [132,133] or could prevent outgrowth of other bacteria in the microbiota [96].

## 5. Conclusions

Recent studies are starting to elucidate the influence of differences in microbiota composition on numerous human disease pathologies. Early results seem to indicate an involvement of HDPs in microbiota composition, potentially creating new opportunities for therapeutic strategies that could help revert disease pathology or prevent further decline in the quality of life of the patient. However, the information on host-microbiota interactions in health and disease is still very limited and future efforts have to focus on development of human relevant platforms to study these interactions accurately and allow discrimination between cause and consequence of changes in microbiota composition, also including the respiratory virome and mycobiome. While a growing number of studies highlight the therapeutic potential of HDPs in the treatment of chronic respiratory diseases, new strategies need to be developed that include studies on human HDP-microbiota interactions in a relevant human environment. These investigations will hopefully establish the exact contribution of HDPs in microbiota composition in the airways and alveoli and help understand the potential role for HDPs in chronic respiratory diseases through modulation of microbiota population. Currently, exciting new developments are ongoing with the use of HDPs designed to express specific immunomodulatory activities, to limit side effects that natural HDPs could display due to their broad-range activities. In animal models, these so-called innate defence regulator peptides (IDRs) were found to limit inflammation and reverse specific features of allergic airway disease [134]. Additionally, studies have been undertaken to identify inducers of endogenous HDPs to promote their expression when their local expression is impaired or otherwise insufficient [135]. Finally, platforms that allow in-depth studies on human host-microbiota interactions that can furthermore elucidate the role of HDPs need to become available. Taken together, these initiatives have the potential to significantly contribute to our knowledge of human host-microbiota interaction in the lungs and its influence on respiratory health and disease.
